# Pulmonary hemodynamic responses to *in utero *ventilation in very immature fetal sheep

**DOI:** 10.1186/1465-9921-11-111

**Published:** 2010-08-19

**Authors:** Beth J Allison, Kelly J Crossley, Sharon J Flecknoe, Colin J Morley, Graeme R Polglase, Stuart B Hooper

**Affiliations:** 1 Dept of Physiology, Monash University, Melbourne, Australia; 2 Neonatal Research, Royal Women's Hospital, Melbourne, Australia; 3 School of Women's and Infants' Health, The University of Western Australia, Perth, Australia; 4 Department of Obstetrics and Gynaecology, Monash Institute of Medical Research, Melbourne, Australia

## Abstract

**Background:**

The onset of ventilation at birth decreases pulmonary vascular resistance (PVR) resulting in a large increase in pulmonary blood flow (PBF). As the large cross sectional area of the pulmonary vascular bed develops late in gestation, we have investigated whether the ventilation-induced increase in PBF is reduced in immature lungs.

**Methods:**

Surgery was performed in fetal sheep at 105 d GA (n = 7; term ~147 d) to insert an endotracheal tube, which was connected to a neonatal ventilation circuit, and a transonic flow probe was placed around the left pulmonary artery. At 110 d GA, fetuses (n = 7) were ventilated *in utero *(IUV) for 12 hrs while continuous measurements of PBF were made, fetuses were allowed to develop *in utero *for a further 7 days following ventilation.

**Results:**

PBF changes were highly variable between animals, increasing from 12.2 ± 6.6 mL/min to a maximum of 78.1 ± 23.1 mL/min in four fetuses after 10 minutes of ventilation. In the remaining three fetuses, little change in PBF was measured in response to IUV. The increases in PBF measured in responding fetuses were not sustained throughout the ventilation period and by 2 hrs of IUV had returned to pre-IUV control values.

**Discussion and conclusion:**

Ventilation of very immature fetal sheep *in utero *increased PBF in 57% of fetuses but this increase was not sustained for more than 2 hrs, despite continuing ventilation. Immature lungs can increase PBF during ventilation, however, the present studies show these changes are transient and highly variable.

## Introduction

Very preterm infants (<28 weeks gestation) are born during the canalicular stage of lung development when the lungs are surfactant-deficient, have a small surface area for gas exchange, a thick air-blood gas barrier and an under-developed pulmonary capillary bed [1]. As a result, very preterm infants commonly suffer respiratory failure after birth and require respiratory support. Although the transition to pulmonary gas exchange is dependent upon major changes in pulmonary hemodynamics, little is known about these changes in infants with very immature lungs and an under-developed pulmonary vascular bed.

During fetal life, pulmonary vascular resistance (PVR) is high and pulmonary blood flow (PBF) is low with most of the blood exiting the right ventricle passing directly into the systemic circulation via the ductus arteriosus (DA). Indeed, pulmonary blood flows are as low as ~10% in fetal sheep and ~24% in human fetuses[2]. The pulmonary arteries develop in parallel with the developing airways [3] and the large cross-sectional surface area of the pulmonary micro-vasculature mainly develops during the saccular and alveolar stages when the distal airways develop [3]. This increase in cross-sectional surface area during late gestation, gradually reduces PVR [4] and is a prerequisite for the large and sustained reduction in PVR after birth at term. This is because, at birth, the pulmonary vascular bed must immediately accept the entire output of the right ventricle, allowing closure of the DA, separation of the pulmonary and systemic circulations and a reduction in pulmonary arterial pressure (PAP). Superimposed on this developmental process is an 8-10 fold reduction in PVR associated with birth, caused by the onset of gaseous ventilation [5-7], increased oxygenation [8,9] and the release of vasodilators [10].

Previous studies on the birth-related changes in pulmonary hemodynamics have focused on the lungs of either mildly preterm or term neonates, all of which were conducted during the alveolar stage of lung development [7,11,12]. During this stage, the alveolar capillary bed is relatively well developed [13] and has a large cross sectional area, giving it the capacity to substantially reduce PVR as ventilation commences. In contrast, in the very immature lung during the canalicular stage, the alveolar vascular bed is still undergoing development and is likely to have a substantially reduced capacity to decrease PVR after birth. However, little is known of the changes in PBF in the very immature lung with the onset of ventilation and so this study has specifically focused on ventilation-induced changes in pulmonary hemodynamics in immature fetal sheep. The results are relevant to understanding pulmonary adaptation at birth in extremely preterm infants.

We have recently developed a technique for ventilating very immature fetal sheep *in utero *[14]. As the fetus remains on placental support, pulmonary ventilation and other aspects of intensive care management are not required to sustain the lamb's viability, allowing it to be ventilated at a stage (110 d gestation) when lung structure closely resembles that of a human infant at 26-28 weeks of gestation. At 110 d of gestation, fetal sheep have few immature alveoli [1] with very few differentiated type-II cells [15] and are not viable if ventilated *ex utero*. Thus, our aim was to examine the effect of *in utero *ventilation (IUV) on pulmonary hemodynamics during the late canalicular stage of lung development in fetal sheep. We hypothesized that the under-developed pulmonary vascular bed would attenuate the increase in PBF associated with the onset of pulmonary ventilation.

## Materials and methods

### Fetal Surgery

**Figure 1:**
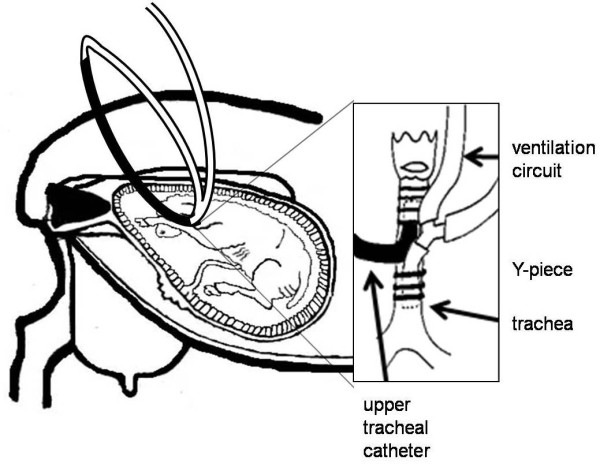
**The ventilation circuit (**white tubes**) was connected to the endotracheal tube via a "Y" piece**. An additional catheter was inserted into the upper trachea (**black tube**) and directed toward the glottis. All catheters were exteriorized via a flank incision. The upper tracheal catheter was connected to the ventilation circuit creating an exteriorized 'tracheal loop' to allow normal flow of lung liquid.

All experimental procedures were approved by the Monash University Animal Ethics Committee. Under general anaesthesia (1.5% halothane in O_2_), aseptic surgery was performed on seven Border-Leicester X Merino ewes at 105 days gestation (term is ~147 days) as previously described [14]. A tube (ID 3.2 mm, OD 6.4 mm) was inserted into the fetal trachea and connected, via a Y-piece, to two large bore, saline-filled, ventilator circuit tubes (ID 9.5 mm, OD 14.3 mm). An additional saline-filled catheter (ID 3.2 mm, OD 6.4 mm) was inserted into the fetal upper trachea and connected to a saline-filled ventilation tube to create an exteriorised tracheal loop, allowing the normal flow of liquid into and out of the lung (Figure 1). A 4-mm ultrasonic flow transducer (Transonic Systems; Ithaca, NY) was placed around the left pulmonary artery (31), this probe directly measures blood flow to the left side of the lung (blood flowing from the right ventricle or shunting left-to-right across the ductus arteriosus). A small securing tie was then inserted into the wall of the vessel and a tapered polyvinyl catheter (BD Insyte Vialon, peripheral venous catheter, length: 48 mm, 0.03 mm ID, 0.041 mm OD, NJ USA) was inserted into the main pulmonary artery, by direct puncture. It was directed 2 cm down into the left pulmonary artery before it was secured into place using the securing tie. Polyvinyl catheters were placed in a fetal carotid artery, jugular vein and amniotic sac and exteriorised via the ewe's flank. Ewes and fetuses were allowed 5 days recovery following surgery. Fetal arterial blood PaO_2_, PaCO_2_, pH and SaO_2 _were measured every second day to assess fetal wellbeing.

### Experimental Protocol

Carotid and pulmonary arterial and amniotic sac pressures (DTX, Viggo-Spectramed, California) and blood flow through the left pulmonary artery (LPBF) were recorded digitally at 1 kHz (Powerlab, ADI: Castle Hill, Australia) for 6 hours prior to starting *in utero *ventilation (IUV). Mean LPBF was calculated electronically from the instantaneous LPBF signal, the LPBF flow measures right ventricular output as well as the left to right shunting through the ductus arteriosus. Prior to IUV, the upper tracheal catheter was disconnected from the ventilator circuit and the liquid within the circuit passively drained by gravity into a sterile bag before connection to a neonatal ventilator (Draeger 8000+). Fetuses were ventilated *in utero *at 110 d gestation for 12 hrs (n = 7) as described previously (briefly PIP of 40 cmH_2_O, PEEP of 4 cmH_2_O, flow 10 L/min and a rate 60 inflations/min; FiO_2 _21%); each fetus acted as its own control. However, operated age-matched control fetuses were also used for comparison of blood-gases (110 d control fetuses) and fetal weight data (117 d control fetuses). Arterial blood gases were measured hourly. Following IUV, the ventilator circuit and fetal lung were refilled with lung liquid and reconnected to the upper tracheal catheter, to restore normal lung liquid flow [14]. Carotid arterial and amniotic sac pressures as well as PBF were recorded digitally for 6 hours after the cessation of IUV and ewes and fetuses were killed 7 days later (117 d GA) for post-mortem analysis.

### Pulmonary Blood Flow Waveform Analysis

Changes in the contour of the PBF waveform were measured by selecting waveforms from 10 consecutive cardiac cycles from each lamb at selected time points during experimentation[11,16]: before IUV (pre-IUV) and then every 5 min for the first two hours of IUV and at 20 min intervals for the remaining 10 hours of IUV. Waveform parameters and calculations of pulsatility index (PI) have been described previously [11].

### Statistical Analysis

PBF and PAP measurements represent an average value taken over a one minute period of recording with care being taken to avoid periods containing obvious movement artefacts caused by the ewe. Measurements of PBF, systemic arterial pressure (SAP) and PAP are expressed as mean ± SEM. Heart rate is expressed a percentage increase from the mean heart rate during the pre-IUV recording period. All values were then grouped and means and standard errors determined. Comparisons of PBF, HR, SAP, PAP and individual components of the PBF waveform were analysed using a two-way repeated measures ANOVA using the statistical package Sigma Stat (Version 3.1.1, Jandel Corporation, USA). The level of significance was p < 0.05 for all statistical analyses.

## Results

### Fetal outcomes

**Figure 2:**
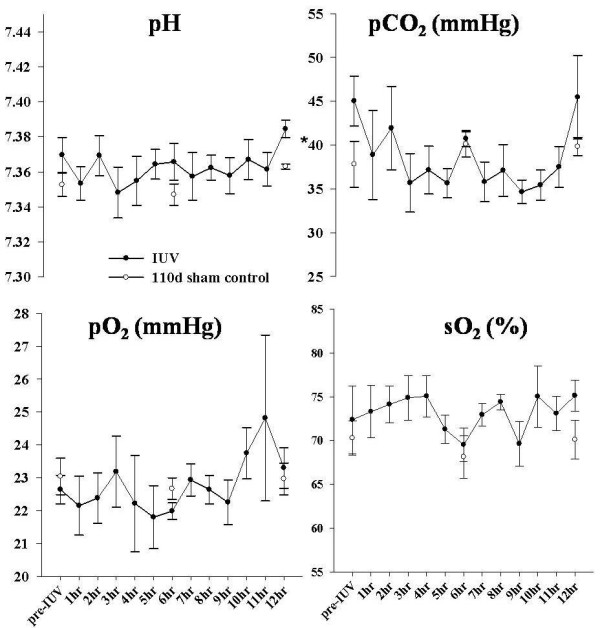
**Fetal blood gas status before and during *in utero *ventilation (IUV)**. Graphs show the Mean ± SEM for pH, PaCO_2_, PaO_2 _and SaO_2 _for fetuses before and during 12 hrs of in utero ventilation (n = 7; black circles). Values are compared to 110 d fetuses (sham operated controls completed for previous study n = 5; white circles). Asterisks indicate statistical difference between IUV and control fetuses.

All fetuses had normal blood gas and acid-base status throughout the experimental period. Although all values were within normal ranges, the pH values in IUV fetuses were significantly higher than in age-matched controls (Figure 2). No time dependent differences were observed in blood gas and acid base status when compared to age-matched controls. No significant differences were observed between fetal body weights and lung/body weight ratios at post mortem compared to age-matched controls (117 d control; Table 1).

**Table 1:** Fetal body and wet lung weights corrected for body weight (mean ± SEM).

			Subgroups	
	**117 d control**	**12 hr IUV + 7 d**	**Responders**	**Non-responders**

GA (days)	117 ± 1	117 ± 1	117 ± 1	116 ± 1

N	5	7	4	3

Body weight (kg)	2.3 ± 0.3	2.3 **± **0.3	1.9 **± **0.1	2.8 **± **0.4

Lung weight (g/kg bw)	33.6 ± 5.6	64.3 **± **15.4	76.9 **± **40.2	51.6 **± **24.7

No statistical differences were present between fetuses that either responded or did not respond to IUV. All measurements were made at the time of post mortem.

### Pulmonary blood flow

**Figure 3:**
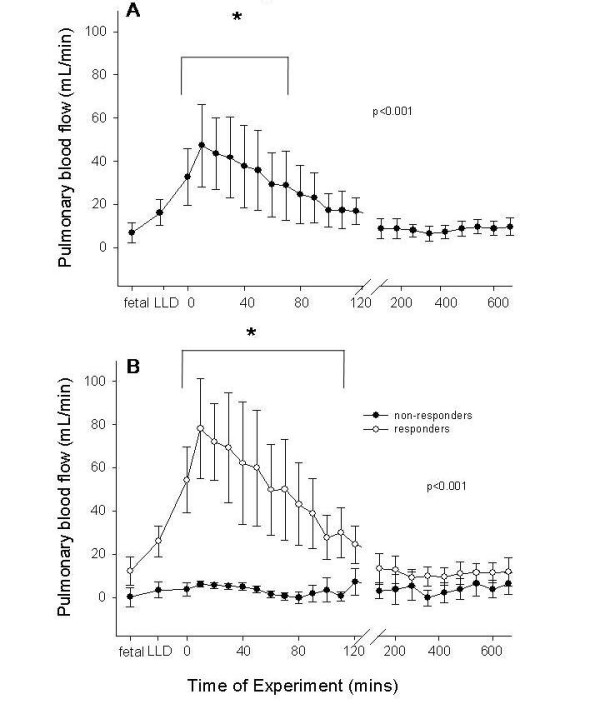
**Changes in pulmonary blood flow (PBF) with *in utero *ventilation (IUV)**. **A**) PBF (mL/min) measured in the left pulmonary artery of IUV exposed fetuses (n = 7) pre-IUV, following lung liquid drainage (LLD) and throughout IUV. Asterisks indicate values which are significantly different from pre-IUV values (p < 0.001). **B**) PBF (mL/min) measured in the left pulmonary artery after sub-division of fetuses into responders (open circles, n = 4) and non-responders (closed circles, n = 3); values were measured pre-IUV, following lung liquid drainage (LLD) and throughout IUV. Asterisks indicate values which are significantly different between responding and non-responding fetuses (p < 0.001).

PBF in the left pulmonary artery tended to increase from pre-IUV control values of 6.9 ± 4.7 mL/min to 16.3 ± 6.1 mL/min in response to lung liquid drainage, although this was not statistically significant. The onset of IUV further, and significantly (p < 0.001), increased PBF from 16.3 ± 6.1 mL/min to a maximum value of 47.2 ± 19.1 mL/min at 10 minutes of IUV (Figure 3A). The PBF then decreased, despite continued mechanical ventilation, such that at 75 minutes after beginning IUV, PBF was not significantly different from pre-IUV values.

**Figure 4:**
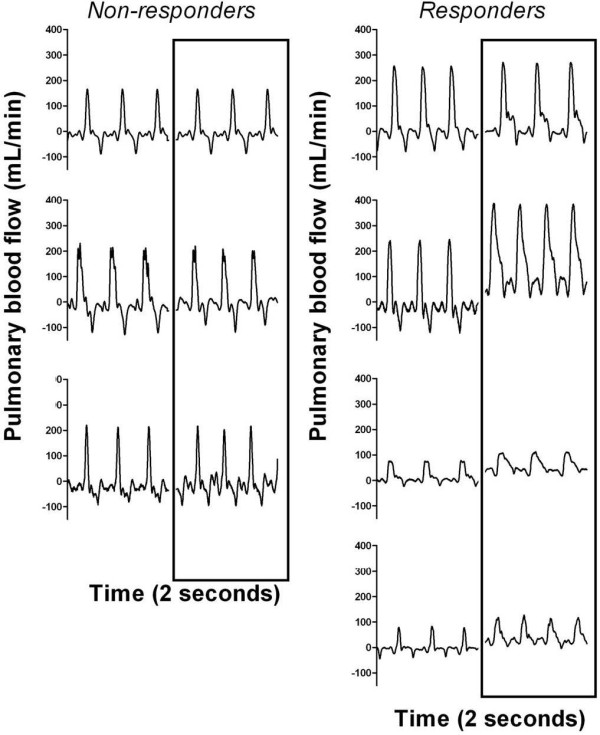
**Pulmonary blood flow (PBF) waveforms**. Individual PBF waveforms are shown for non-responding (left) and responding (right) fetuses. Two sets of waveforms are displayed the first taken during the fetal period and the second during *in utero *ventilation (enclosed in the square).

The change in PBF in response to IUV varied markedly between fetuses, particularly during the first 90 minutes of IUV, explaining the high variability in PBF displayed in Figure 3A. Based upon the PBF response to IUV, individual fetuses have been subdivided into two groups which differed markedly in their PBF response (Figure 3B). Group (i) called "*responders" *includes all fetuses with an end-diastolic flow >0 ml/min (indicating retrograde flow from the pulmonary arteries had ceased; see Figure 4) and a >50% increase in PBF (above control values) (Figure 3B; *responders*). Group (ii) called "*non-responders"*, were fetuses who displayed minor or no alterations in PBF (Figure 3B, *non-responders*), including little change in end-diastolic flow (Figure 4). Of the seven fetuses studied, 4 were *responders *and 3 *non-responders*. GA, body weights and lung weights (Table 1) were not different between the groups.

In comparison to non-responding fetuses, responding fetuses markedly increased PBF in response to IUV, increasing from a pre-IUV value of 12.2 ± 6.6 mL/min to a maximum of 78.1 ± 23.1 mL/min at 10 minutes of IUV after which PBF gradually decreased to return to fetal levels at 2 hours of IUV (Figure 3B). Although all fetuses increased PBF in response to IUV in this group, the extent of the increase varied between fetuses during the early stages of IUV. In contrast, no change in PBF occurred in *non-responding *fetuses throughout the IUV period (Figure 3B). The changes in PBF were significantly different between responding and non-responding fetuses (p < 0.001) from the beginning until 110 mins of mechanical ventilation.

### PBF waveform analysis

Numerous transient changes in PBF waveform characteristics were noted, although when both groups were combined, only the increases in mean diastolic flow and post-systolic minimum flow at 20 mins of IUV as well as the decrease in pulsatility index at 20 minutes and 2 hours were significant (Table 2). The inability to detect significant changes was largely caused by non-responding fetuses, as they had little or no variation in their PBF waveform (Figure 4; left hand graphs) in response to IUV. In contrast, fetuses that responded to IUV had markedly altered PBF waveforms (Figure 4; right hand graphs) that were similar to what we have shown in more mature fetuses [17].

**Table 2:** Characteristics of the pulmonary blood flow (PBF) waveform in fetuses before, during (at selected time points) and after *in utero *ventilation (IUV).

		*In Utero *Ventilation			
	**Pre-IUV**	**20 min**	**2 hr**	**11 hr**	**Post-IUV**

**Peak systolic flow (mL/min)**	189.5 ± 30.5	228.4 ± 42.2	179.3 ± 32.3	177.0 ± 37.3	189.0 ± 34.9

**Post-systolic minimum flow (mL/min)**	-78.6 ± 13.9^**a**^	-32.5 ± 22.8^**b**^	-68.8 ± 13.4^**a**^	-80.8 ± 13.2^**a**^	-75.4 ± 15.4^**a**^

**End-diastolic flow (mL/min)**	-27.1 ± 4.4	-10.1 ± 13.9	-26.0 ± 6.5	-30.7 ± 7.2	-26.1 ± 0.3

**Mean diastolic flow (mL/min)**	-17.7 ± 4.4^**a**^	18.4 ±2.2^**b**^	-12.6 ± 3.7^**a**^	-14.1 ± 3.0^**a**^	-9.6 ± 5.1^**a**^

**Pulsatility index**	1.4 ± 0.0^**a**^	1.2 ± 0.1^**b**^	1.1 ± 0.1^**b**^	1.47 ± 0.0^**a**^	1.40 ± 0.0^**a**^

**Pulse amplitude (mL/min)**	207.8 ± 31.4	173.1 ± 37.2	188.4 ± 28.8	167.1 ± 34.1	213.1 ± 42.8

Values that do not share a common letter are significantly different from each other (p< 0.05).

As we have shown previously during preterm ventilation [17], IUV alter diastolic characteristics of the PBF waveform. Most changes were transient and paralleled changes in mean PBF (Table 3). In responding fetuses, mean diastolic flow, post-systolic minimum flow and end-diastolic flow were all significantly increased (Table 3; p < 0.05) from pre-IUV values at 20 minutes of IUV and returned to baseline levels by 2 hrs of ventilation. In 3 out of the 4 responding fetuses, PBF displayed no retrograde flow. PI significantly (p < 0.05), but transiently, decreased in responding fetuses (Table 3). Overall in all parameters measured, the responding fetuses were significantly different to the non-responding fetuses (p < 0.001).

**Table 3:** Characteristics of the pulmonary blood flow (PBF) waveform in fetuses before during (at selected time points) and after *in utero *ventilation (IUV).

	RESPONDERS					NON RESPONDERS				
	**PRE-IUV**	**20 min**	**2 hrs**	**11 hrs**	**POST IUV**	**PRE-IUV**	**20 min**	**2 hrs**	**11 hrs**	**POST-IUV**

**Peak systolic flow (mL/min)**	179 ± 54^**a**^	234 ± 65^**b**^	173 ± 54^**a**^	174 ± 54^**a**^	159 ± 46^**a**^	203 ± 19^**xy**^	220 ± 14^**xy**^	187 ± 36^**x**^	181 ± 33^**xy**^	247 ± 1^**x**^

**Post-systolic minimum flow (mL/min)**	-65.9 ± 22	11.5 ± 14	-52.9 ± 12	-75.0 ± 19	-66.2 ± 20	-71.6 ± 11	-90.3 ± 14	-89.8 ± 24	-88.5 ± 21	-94.0 ± 16

**End-diastolic flow (mL)**	-22.6 ± 6.5^**a**^	11.5 ± 10.9^**b**^	-19.1 ± 5.0^**a**^	-28.1 ± 8.8^**a**^	-24.2 ± 7.4^**a**^	-33.2 ± 1.3	-38.8 ± 0.7	-35.3 ± 13.1	-34.1 ± 9.6	-29.9 ± 5.6

**Mean diastolic flow (mL)**	-10.9 ± 4.7^**a**^	49.0 ± 16.5^**b**^	-6.1 ± 2.7^**a**^	-15.3 ± 2.3^**a**^	-15.4 ± 4.7^**a**^	-26.8 ± 4.0	-22.4 ± 5.2	-21.2 ± 4.2	-15.7 ± 6.2	-17.7 ± 10.7

**Pulsatility index**	1.4 ± 0.0^**a**^	1.0 ± 0.0^**b**^	1.0 ± 0.1^**b**^	1.5 ± 0.0^**a**^	1.4 ± 0.0^**a**^	1.5 ± 0.0	1.4 ± 0.0	1.2 ± 0.2	1.5 ± 0.0	1.4 ± 0.1

**Pulse amplitude (mL/min)**	193 ± 54	162 ± 34	167 ± 47	170 ± 44	165 ± 48	227 ± 24	230 ± 15	216 ± 46	212 ± 35	242 ± 2

Values that do not share a common letter are significantly different from each other (p< 0.05). Overall in all parameters measured, the responding fetuses were significantly different to the non-responding fetuses (p<0.001).

### Heart rate

**Figure 5:**
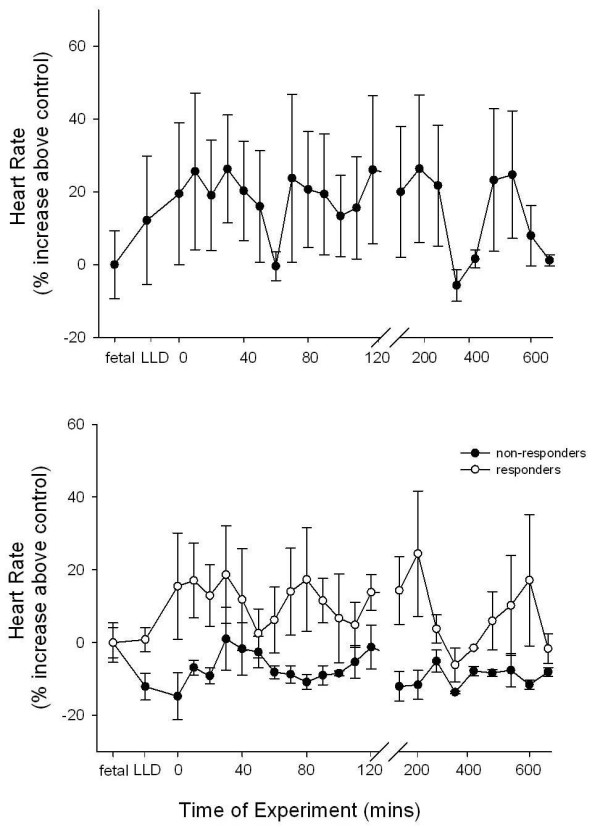
**Changes in heart rate with *in utero *ventilation (IUV)**. **A)** Values represent % increase in heart rate above control values of IUV exposed fetuses (n = 7) pre-IUV, following lung liquid drainage (LLD) and throughout IUV. **B)** Values represent % increase in heart rate above control values in responding (open circles, n = 4) and non-responders (closed circles, n = 3) IUV fetuses; values were measured pre-IUV, following lung liquid drainage (LLD) and throughout IUV.

Fetal heart rate tended to increase within 30 mins of IUV, although this increase was not significant either when the groups were considered together (Figure 5A) or following separation into responders and non responders (Figure 5B) due to the high level of variability.

### Pulmonary and systemic arterial pressure

Both the PAP and SAP were not significantly altered throughout IUV (Table 4). Following subdivision into groups based on their PBF response to IUV, neither pulmonary nor systemic arterial pressures were significantly altered by IUV (subgroup data not shown).

**Table 4:** Pulmonary and systemic arterial pressures in fetuses before during (at selected time points) and after *in utero *ventilation (IUV).

	RESPONDERS							NON RESPONDERS				
	**PRE-IUV**	**20 min**	**2 hrs**	**11 hrs**	**POST-IUV**			**PRE-IUV**	**20 min**	**2 hrs**	**11 hrs**	**POST-IUV**

**Pulmonary Arterial ** **Pressure (mmHg)**	37.9 ± 1.8	36.8 ± 2.0	38.0 ± 2.5	36.8 ± 3.0	40.0 ± 1.0			38.7 ± 3.5	31.1 ± 11.1	39.7 ± 2.0	40.0 ± 0.0	32.5 ± 6.7

**Systemic Arterial ** **Pressure (mmHg)**	37.4 ± 3.2	38.0 ± 4.3	39.5 ± 4.4	41.9 ± 3.0	43.0 ± 2.1			37.7 ± 3.9	36.2 ± 5.0	38.4 ± 3.0	36.5 ± 3.4	36.7 ± 2.8

## Discussion

It is known that ventilation after birth rapidly reduces PVR and increases PBF in mature fetuses at term [7], but how the very immature fetus responds to ventilation is unknown. This study has shown that mechanical ventilation of extremely immature fetal sheep *in utero *transiently increased PBF in a subgroup of fetuses, although even these fetuses were unable to sustain this increase for longer than 2 hours, despite continuing mechanical ventilation. In the other fetuses, PBF did not increase at any stage during the mechanical ventilation period. The cause of the large variability in pulmonary hemodynamic responses to mechanical ventilation between fetuses remains unknown, although fetal heart rate was also variable. This study is the first to demonstrate the variable PBF response to mechanical ventilation in extremely immature lambs and shows that although the PBF increase is rapid in some lambs, when present it is transient, lasting <2 hrs.

### Alterations to Pulmonary Hemodynamics with IUV

Previous studies have shown that reducing lung liquid volumes in near term fetal sheep, to a volume equivalent to FRC in newborn lambs, decreases PVR and increases PBF 2-4 fold [18,19]. Similarly, previous IUV studies conducted in near term fetal sheep have shown that IUV causes a sustained decrease in PVR and increase in PBF [7,20,21]. In this study, lung liquid drainage prior to IUV caused only a small increase PBF, which failed to reach statistical significance, and mechanical ventilation caused a variable increase in PBF in only ~57% of fetuses. Clearly, the difference between the findings of this study and previous studies is the gestational age and lung maturity at the time of lung liquid drainage and IUV. This suggests that in very preterm infants, with a similar level of lung immaturity, the capacity of the lung to increase PBF in response to mechanical ventilation may be considerably less than previously acknowledged and non-existent in some.

The mechanisms responsible for the increase in PBF at birth in mature, near term, fetuses are thought to be multi-factorial and include effects of ventilation, increased oxygenation and the release of vasodilators [22-24]. NO-induced vasodilation is thought to contribute to ~50% of the increase in PBF at birth [22] which can be inhibited by blocking NO activity [25]. The mature fetal lung can also vasodilate in response to vasoactive factors, such as prostaglandin D_2 _[26] and bradykinin [27] both of which can decrease PVR and release vasoactive substances, such as prostaglandins in response to rhythmic distension [28,29]. However, the ability of the very immature pulmonary vascular bed to release NO and other vasodilators and respond to them is relatively unexplored and warrants further investigation.

Changes in transpulmonary pressures associated with lung aeration also contributes to the increase in PBF with ventilation onset [7,30,31]. Before birth, the fetal lungs are liquid-filled providing a constant internal distending pressure that maintains the lungs in an expanded state [32,33]. Following lung aeration, the distending influence of lung liquid is lost and the presence of air creates an air-liquid interface [32]. The resulting surface tension, even in the presence of surfactant, increases lung recoil and creates sub-atmospheric intra-pleural and peri-alveolar interstitial tissue pressures [34,35]. This increases both the capillary/alveolar wall and capillary/interstitial tissue wall transmural pressures leading to an increase in capillary recruitment [36] and expansion of recruited capillaries [31]. On the other hand, increases in intra-luminal pressure caused by positive pressure ventilation, reduces the alveolar/capillary transmural pressure, capillary calibre and increases PVR. This is a well established relationship that has been demonstrated in adults [37], near term fetuses [7], newborns [38] and relatively mature preterm lambs [11].

In adults, the large cross-sectional area of the pulmonary capillary bed allows the pulmonary circulation to accept the entire output of the right ventricle while maintaining pressures at ~1/8 of the systemic circulation. Much of the capillary bed forms (and the cross-sectional area of bed increases) during the alveolar stage of lung development, resulting in a gradual reduction in PVR during late gestation [39]. At birth the large decrease in PVR can only occur if the cross-sectional area of the pulmonary vascular bed is sufficiently large. As very immature lungs will not have undergone this vital development at the time of preterm birth [40] the ability of the immature lung to decrease PVR after birth must be diminished, irrespective of whether they have the capacity to dilate in response to vasodilatory stimuli. Indeed the decrease Pulsatility Index (PI) (Table 2 and 3) in fetal sheep following the initiation of mechanical ventilation in the current study indicates that PVR did decrease, although again the increased PI was not maintained for longer than 2 hours. The lack of a pulmonary vascular bed with a sufficiently high cross sectional area may explain why the increase in PBF was not sustained in our study and only occurred in 57% of fetuses.

The contribution of the low resistance placental circulation must also be considered when interpreting PBF changes in response to IUV, as the placental circulation was functional during our studies. We have recently shown that flow through the ductus arteriosus (DA) reverses with mechanical ventilation at birth, due to a reversal of the pressure gradient across the DA [16]. As a result, the majority of blood flow through the DA immediately after birth, flows from the systemic into the pulmonary circulation (referred to as left-to-right shunting) which contributes ~50% of PBF that gradually decreases with time [16]. The reversal of the pressure gradient between the systemic and pulmonary circulations [16,19] and the onset of left-to-right shunting through the DA, must be determined by both the decrease in PVR and the increase in downstream resistance in the peripheral vascular bed caused by umbilical cord occlusion. As the umbilical cord was not occluded in this study resistance in the systemic circulation would have remained low limiting the change in pressure gradient across the DA.

Heart rate in the responders, although tending to increase, was not significantly altered throughout the experiment. Given that fetal cardiac output is predominately determined by heart rate rather than stroke volume [41], it is possible that right ventricular output may have contributed to the initial increase in PBF. However, the decrease in PVR (as determined by the pulsatility index) likely plays a greater role in the present study as previously discussed. However, the increase in PBF is likely to have a combination of right and left ventricular output contributions. The responders had positive PBF throughout the cardiac cycle, evident by their respective PBF waveforms (Figure 4), indicating that 100% of right ventricular output is entering the pulmonary circulation. Left ventricular output may contribute to the increase in PBF as evident by the decrease in the PI (Table 4) which would facilitate left-to-right shunting through the DA. The gradual increase in pulsatility index, likely caused by factors which shall be discussed later, would reduce both left and right ventricular output contributions thus decreasing PBF to normal levels.

### The Effect of IUV on Pulmonary Blood Flow Waveform Contour

In responding fetuses, the contour of the PBF waveform changed in response to IUV to closely resemble the waveform seen in post-natal animals [17], with flow during diastole being particularly affected (Figure 4). Specifically, IUV significantly increased end- and peak-systolic flow as well as end- and mean-diastolic flow, resulting in decreased pulse amplitude in the early stages of IUV. The most significant change was the increase in diastolic flow about 20 mins after mechanical ventilation onset where it remained positive in 3 out of the 4 responding fetuses, indicating blood flowed towards the lung throughout the cardiac cycle at this time. The loss of retrograde flow during diastole is the greatest change to the PBF waveform during fetal to neonatal transition and is a sensitive indicator of a reduced PVR [11]. Thus, the loss of retrograde flow in these very immature fetuses implies that PVR was reduced and that some left-to-right shunting through the DA may have occurred which contributed to the increase in PBF [16]. As diastolic PBF did not increase in non-responding fetuses, it is unlikely that PVR was significantly reduced or that significant levels of left-to-right shunting occurred in these fetuses in response to IUV.

### Other differences between responding and non-responding fetuses

The two fetal sub-groups had similar arterial pressures and blood gas status before and during IUV, and at autopsy had similar body and organ weights. However, in addition to the PBF changes, responding fetuses increased their heart rate in response to IUV. Although this may have contributed to the increase in PBF, it cannot account for the differences in PBF between subgroups. Indeed, one of the largest changes in PBF in response to IUV was increased diastolic flow, which is largely independent of heart rate and mostly determined by PVR [11]. An increase in fetal heart rate, in the absence of a decrease in PVR, simply results in increased right-to-left flow through the DA. Previous IUV studies conducted in near term fetuses [9,12], also found that maximal increases in PBF occurred in only half of ventilated fetuses [7,42]. Teitel and colleagues (1990) also separated fetuses into major and minor responders (determined by PBF changes) but found no differences between groups other than their response to IUV; they looked at gender, fetal weight, blood gas status, pulmonary vascular tone, ventricular function and alveolar ventilation [7]. We consider the primary difference between responding and non-responding fetuses is the effect of IUV on PVR.

It is also possible that fetal posture and the influence of the surrounding amniotic fluid may play a role in the differential PBF responses to IUV. Fetal body position cannot be held constant during IUV, either between animals or throughout experimentation in individual animals. Increased fetal flexion is known to increase abdominal pressure and elevate the fetal diaphragm [43] which could limit the tidal volume and distribution of ventilation within the fetal lung. The net result would likely be a marked reduction in the ventilation-induced increase in PBF.

### Transient Nature of Hemodynamic Changes

Despite the period of IUV lasting for 12 hours, the increase in PBF only lasted ~40 minutes in responding fetuses. Transient PBF changes in response to IUV have not been reported previously although this may be because ventilation only lasted for 30 min or less in those studies [7-9,42]. Attenuation of increases in PBF have been described in fetuses following occlusion of the DA [44], exposure to high levels of inspired oxygen [45] and to vasodilators [46]. Abman and Accurso (1989) have suggested that the immature vascular bed may have a limited ability to release and maintain the necessary vasodilatory factors needed to sustain an increase in PBF. Alternatively, it is possible that PVR progressively increases, after an initial decrease, due to compression of the capillaries from increased interstitial tissue pressure caused by lung liquid retention in the tissue [35] or to oedema caused by injury [47]. It is also possible that an increase in vasoconstrictive properties within the vascular bed caused by neural, humoral, local or a myogenic responses contributes to the transient nature for the increase in PBF [44]. Shear stress associated with mechanical ventilation is known to elicit myogenic responses [48] which the pulmonary vasculature is particularly sensitive to during the perinatal period [49].

In summary, our studies indicate that despite significant structural immaturity, the very immature lung can increase PBF in response to mechanical ventilation although changes are transient and highly variable. As previous studies, conducted *ex utero*, have not demonstrated the same variability this suggests either the degree of lung immaturity or the *in utero *environment is associated with the variability. Understanding the changes in pulmonary hemodynamics at birth in the extremely immature lung is necessary to improve the care and management of extremely preterm infants.

## Abbreviations

DA: Ductus arteriosus; GA: Gestational age; IUV: *In utero *ventilation; LPBF: Left pulmonary arterial blood flow; PAP: Pulmonary arterial pressure; PBF: Pulmonary blood flow; PI: Pulsatility index; PVR: Pulmonary vascular resistance; SAP: Systemic arterial pressure

## Competing interests

The authors declare that they have no competing interests.

## Authors' contributions

BA participated in designing the experiments, carried out experiments, analysis of data and drafting of the manuscript. KC assisted with experiments and assisted in drafting of the manuscript. SF assisted with experiments and drafting of the manuscript. CM assisted with the analysis of data and drafting of the manuscript. GP assisted with the analysis of data and drafting of the manuscript. SH participated in designing the experiments, assisted with experiments, analysis of data and drafting of the manuscript. All authors read and approved the final manuscript.

## References

[B1] Alcorn DG Adamson TM Maloney JE Robinson PM A morphologic and morphometric analysis of fetal lung development in the sheep Anat Rec 1981 201 655 667 10.1002/ar.1092010410 7340570

[B2] Rasanen J Huhta JC Weiner S Wood DC Ludomirski A Fetal branch pulmonary arterial vascular impedance during the second half of pregnancy Am J Obstet Gynecol 1996 174 1441 1449 10.1016/S0002-9378(96)70586-0 9065109

[B3] Hislop A Reid L Intra-pulmonary arterial development during fetal life - branching pattern and structure J Anat 1972 113 35 48 4648482 PMC1271365

[B4] Rudolph AM Heymann MA Control of the Foetal circulation Foetal and neonatal physiology Proceedings of the Sir Joseph Barcroft centenary symposium The Physiological Laboratory Cambridge 25 to 27 of july 1972 1973 London: Cambridge University Press 89 111

[B5] Dawes GS MILNE ED Mott JC Widdicombe JG The closure of the foramen ovale after birth J Physiol 1953 122 38P 13109794

[B6] Heymann MA Control of the pulmonary circulation in the fetus and during the transitional period to air breathing Obstet Gynecol 1999 84 127 132 10.1016/s0301-2115(98)00321-2 10428335

[B7] Teitel DF Iwamoto HS Rudolph AM Changes in the pulmonary circulation during birth-related events Pediatr Res 1990 27 372 378 10.1203/00006450-199004000-00010 2342829

[B8] Banerjee A Roman C Heymann MA Bradykinin receptor blockade does not affect oxygen-mediated pulmonary vasodilation in fetal lambs Pediatr Res 1994 36 474 480 10.1203/00006450-199410000-00010 7816523

[B9] Moore P Velvis H Fineman JR Soifer SJ Heymann MA EDRF inhibition attenuates the increase in pulmonary blood flow due to oxygen ventilation in fetal lambs J Appl Physiol 1992 73 2151 2157 1474097 10.1152/jappl.1992.73.5.2151

[B10] Abman SH Chatfield BA Hall SL McMurtry IF Role of endothelium-derived relaxing factor during transition of pulmonary circulation at birth Am J Physiol 1990 259 H1921 H1927 2260716 10.1152/ajpheart.1990.259.6.H1921

[B11] Polglase GR Morley CJ Crossley KJ Dargaville P Harding R Morgan DL Hooper SB Positive end-expiratory pressure differentially alters pulmonary hemodynamics and oxygenation in ventilated, very premature lambs J Appl Physiol 2005 99 1453 1461 10.1152/japplphysiol.00055.2005 15890759

[B12] Morin FCI Egan EA Pulmonary hemodynamics in fetal lambs during development at normal and increased oxygen tension J Appl Physiol 1992 73 213 218 1506372 10.1152/jappl.1992.73.1.213

[B13] Levin DL Rudolph AM Heymann MA Phibbs RH Morphological development of the pulmonary vascular bed in fetal lambs Circulation 1976 53 144 151 1244236 10.1161/01.cir.53.1.144

[B14] Allison BJ Crossley KJ Flecknoe SJ Davis PG Morley CJ Harding R Hooper SB Ventilation of the very immature lung in utero induces injury and BPD-like changes in lung structure in fetal sheep Pediatr Res 2008 64 387 392 10.1203/PDR.0b013e318181e05e 18552709

[B15] Flecknoe SJ Wallace MJ Cock ML Harding R Hooper SB Changes in alveolar epithelial cell proportions during fetal and postnatal development in sheep Am J Physiol Lung Cell Mol Physiol 2003 285 L664 L670 12794005 10.1152/ajplung.00306.2002

[B16] Crossley KJ Allison BJ Polglase GR Morley CJ Davis PG Hooper SB Dynamic changes in the direction of blood flow through the ductus arteriosus at birth J Physiol-London 2009 587 Pt 19 4695 4703 10.1113/jphysiol.2009.174870 19675069 PMC2768022

[B17] Polglase GR Wallace MJ Morgan DL Hooper SB Increases in lung expansion alter pulmonary hemodynamics in fetal sheep J Appl Physiol 2006 101 273 282 10.1152/japplphysiol.01544.2005 16575019

[B18] Walker AM Ritchie BC Adamson TM Maloney JE Effect of changing lung liquid volume on the pulmonary circulation of fetal lambs J Appl Physiol 1988 64 61 67 3356667 10.1152/jappl.1988.64.1.61

[B19] Hooper SB Role of luminal volume changes in the increase in pulmonary blood flow at birth in sheep Exp Physiol 1998 83 833 842 9782192 10.1113/expphysiol.1998.sp004163

[B20] Rudolph AM Heymann MA Hemodynamic changes induced by blockers of prostaglandin synthesis in the fetal lamb in utero Adv Prostaglandin Thromboxane Res 1978 4 231 237 417569

[B21] Reid DL Thornburg KL Pulmonary pressure-flow relationships in the fetal lamb during in utero ventilation J Appl Physiol 1990 69 1630 1636 2272954 10.1152/jappl.1990.69.5.1630

[B22] Rairigh RL Parker TA Ivy DD Kinsella JP Fan ID Abman SH Role of inducible nitric oxide synthase in the pulmonary vascular response to birth-related stimuli in the ovine fetus Circ Res 2001 88 721 726 10.1161/hh0701.088683 11304495

[B23] Abman SH Harding R, Pinkerton KE, Plopper CG Developmental physiology of the pulmonary circulation The Lung: Development, Aging and the Environment 2004 Oxford: Butterworth Heinemann 105 117

[B24] Leffler CW Hessler JR Green RS Mechanism of stimulation of pulmonary prostacyclin synthesis at birth Prostaglandins 1984 28 877 887 10.1016/0090-6980(84)90041-8 6441191

[B25] Abman SH Chatfield BA Hall SL McMurtry IF Role of endothelium-derived relaxing factor during transition of pulmonary circulation at birth Am J Physiol 1990 259 H1921 H1927 2260716 10.1152/ajpheart.1990.259.6.H1921

[B26] Cassin S Role of prostaglandins and thromboxanes in the control of the pulmonary circulation in the fetus and newborn Semin Perinatol 1980 4 101 107 6990500

[B27] Campbell AGM Cockburn F Dawes GS Milligan JE Pulmonary Vasoconstriction in asphyxia during cross-circulation between twin foetal lambs J Physiol 1967 192 111 121 6069314 10.1113/jphysiol.1967.sp008291 PMC1365476

[B28] Velvis H Moore P Heymann MA Prostaglandin inhibition prevents the fall in pulmonary vascular resistance as a result of rhythmic distension of the lungs in fetal lambs Pediatr Res 1991 30 62 68 1891282 10.1203/00006450-199107000-00013

[B29] Leffler CW Hessler JR Green RS The onset of breathing at birth stimulates pulmonary vascular prostacyclin synthesis Pediatr Res 1984 18 938 942 6387607 10.1203/00006450-198410000-00006

[B30] Hooper SB Kitchen MJ Wallace MJ Yagi N Uesugi K Morgan MJ Hall C Siu KK Williams IM Siew M Imaging lung aeration and lung liquid clearance at birth FASEB J 2007 21 3329 3337 10.1096/fj.07-8208com 17536040

[B31] Hooper SB Harding R Role of aeration in the physiological adaptation of the lung to air-breathing at birth Current Respiratory Medicine Reviews 2005 1 185 195 10.2174/1573398054023037

[B32] Hooper SB Harding R Fetal lung liquid: a major determinant of the growth and functional development of the fetal lung Clin Exp Pharmacol Physiol 1995 22 235 247 10.1111/j.1440-1681.1995.tb01988.x 7671435

[B33] Harding R Hooper SB Regulation of lung expansion and lung growth before birth J Appl Physiol 1996 81 209 224 8828667 10.1152/jappl.1996.81.1.209

[B34] Avery ME Cook CD Volume-pressure relationships of lungs and thorax in fetal, newborn, and adult goats J Appl Physiol 1961 16 1034 1038 13863394 10.1152/jappl.1961.16.6.1034

[B35] Miserocchi G Negrini D Del Fabbro M Venturoli D Pulmonary interstitial pressure in intact in situ lung: transition to interstitial edema J Appl Physiol 1993 74 1171 1177 8482655 10.1152/jappl.1993.74.3.1171

[B36] Lipsett J Hunt K Carati C Gannon B Changes in the spatial distribution of pulmonary blood flow during the fetal/neonatal transition: an in vivo study in the rabbit Pediatr Pulmonol 1989 6 213 222 10.1002/ppul.1950060402 2748216

[B37] Olson LE Wardle RL Effect of a regional increase in alveolar pressure on pulmonary blood flow J Appl Physiol 1992 73 1291 1296 1447072 10.1152/jappl.1992.73.4.1291

[B38] Fuhrman BP Smith-Wright DL Kulik TJ Lock JE Effects of static and fluctuating airway pressure on intact pulmonary circulation J Appl Physiol 1986 60 114 122 3511021 10.1152/jappl.1986.60.1.114

[B39] Rudolph AM Fetal and neonatal pulmonary circulation Annu Rev Physiol 1979 41 383 395 10.1146/annurev.ph.41.030179.002123 35091

[B40] Reid L Meyrick B Microcirculation: definition and organization at tissue level Ann N Y Acad Sci 1982 384 3 20 10.1111/j.1749-6632.1982.tb21357.x 6953826

[B41] Rudolph AM Heymann MA Cardiac output in the fetal lamb: The effects of spontaneous and induced changes of heart rate on right and left ventricular output Am J Obstet Gynecol 1976 124 183 192 129010 10.1016/s0002-9378(16)33296-3

[B42] Iwamoto HS Teitel DF Rudolph AM Effects of lung distension and spontaneous fetal breathing on hemodynamics in sheep Pediatr Res 1993 33 639 644 10.1203/00006450-199306000-00021 8378125

[B43] Harding R Hooper SB Dickson KA A mechanism leading to reduced lung expansion and lung hypoplasia in fetal sheep during oligohydramnios Am J Obstet Gynecol 1990 163 1904 1913 2256503 10.1016/0002-9378(90)90772-y

[B44] Abman SH Accurso FJ Acute effects of partial compression of ductus arteriosus on fetal pulmonary circulation Am J Physiol 1989 257 H626 H634 2764143 10.1152/ajpheart.1989.257.2.H626

[B45] Accurso FJ Alpert B Wilkening RB Peterson RG Meschia G Time-dependent response of fetal pulmonary blood flow to an increase in fetal oxygen tension Respir Physiol 1986 63 43 52 10.1016/0034-5687(86)90029-0 3081980

[B46] Accurso FJ Wilkening RB Temporal response of the fetal pulmonary circulation to pharmacologic vasodilators Proc Soc Exp Biol Med 1988 187 89 98 3340622 10.3181/00379727-187-42642

[B47] Schuster DP Haller J A quantitative correlation of extravascular lung water accumulation with vascular-permeability and hydrostatic-pressure measurements - apositron emission tomography study Journal of Critical Care 1990 5 161 168 10.1016/0883-9441(90)90037-A

[B48] Storme L Rairigh RL Parker TA Kinsella JP Abman SH In vivo evidence for a myogenic response in the fetal pulmonary circulation Pediatr Res 1999 45 425 431 10.1203/00006450-199903000-00022 10088665

[B49] Belik J Keenley FW Baldwin F Rabinovitch M Pulmonary hypertension and vascular remodeling in fetal sheep Am J Physiol 1994 266 h2303 h2309 8023991 10.1152/ajpheart.1994.266.6.H2303

